# Auditory skill analysis of a group of subjects with history of illicit drug use

**DOI:** 10.1016/S1808-8694(15)30519-X

**Published:** 2015-10-18

**Authors:** Daniela de Lucas Rosseto, Silvana Cristina Ribeiro, Mônica Pires de Castro Mendonça, José Antônio A. de Oliveira, Ana Cláudia Mirândola Barbosa Reis, Sinésio Grace Dutra

**Affiliations:** 1 Speech and Hearing Therapist, Student at the Specialization Program on Audiology, Franca University; 2 Speech and Hearing Therapist, Student at the Specialization Program on Audiology, Franca University - Unifran; 3 PhD student at the Department of Ophthalmology, Otorhinolaryngology, and Head/Neck Surgery at the Medical School of Ribeirão Preto/SP-USP. Professor of Speech and Hearing Therapy Programa t Franca University - Unifran; 4 Professor / PhD, Professor at the Department of Ophthalmology, Otorhinolaryngology, and Head/Neck Surgery at the Medical School of Ribeirão Preto/SP-USP; 5 PhD on Human Communication Disorders: Speech and Hearing Therapist at Unifesp/EPM. Professor at the Speech and Hearing Therapy Program, Department of Ophthalmology, Otorhinolaryngology, and Head/Neck Surgery at the Medical School of Ribeirão Preto/SP-USP; 6 PhD on General Surgery at the Medical School of Ribeirão Preto/SP-USP. Neurosurgeon. Professor at the Speech and Hearing Therapy Program - Franca University - Unifran. Franca University and Graduate Program of the Department of Ophthalmology, Otorhinolaryngology, and Head/Neck Surgery at the Medical School of Ribeirão Preto/SP-USP

**Keywords:** attention, hearing, auditory cortex, drugs

## Abstract

Illicit drugs affect the central nervous system by introducing alterations in cognitive, attention and memory functions.

**Aim:**

this paper aims to characterize the auditory selective attention skills of subjects with history of illicit drug use and check whether the amount of time for which these subjects took drugs impacts the severity of the encountered alterations.

**Materials and method:**

this is a cohort, cross-sectional retrospective study. Nineteen male subjects with history of drug use and ages ranging between 16 and 47 years were analyzed. Statistical test: ‘Mann-Whitney’.

**Procedure:**

initial interview, ENT examination, audiological examination, auditory processing assessment - Staggered Spondaic Word Test - SSW.

**Results:**

extremely significant statistical differences were found in the number of errors found in the four listening conditions when control and case group findings were compared. However, when case group subject findings were compared, no statistically significant difference was found.

**Conclusion:**

the used auditory processing tests - SSW - were sensitive enough to capture and assist in the diagnosis of alterations introduced by the deleterious impact of drug use upon the CNS. The time for which subjects used drugs is not a determining factor on alteration severity.

## INTRODUCTION

Language plays a primordial role in human development. Anatomic and physiologic integrity of both neurologic and auditory systems is a prerequisite that in combination with intellectual, psychical, and emotional traits, alongside proper stimulation and socialization, allow for adequate language acquisition and development.

Among the functions developed by human beings, hearing is the one that initially is of utter importance in the acquisition and development of verbal communication, as it enables the perception of sound stimuli. Such stimuli are captured by the ear and carried to the corresponding area in the brain, where they are analyzed and interpreted. Sound stimuli interpretation provides meaning to sounds of the environment and speech, turning individuals able to develop communication skills through spoken and written language, greatly enhancing their socialization.^1^,^2^

At birth, the structures pertaining to the auditory system (ears and auditory portions of the cortex) are formed, but hearing will only develop as the child is exposed to sounds. Such exposure fosters the development of auditory processing and speech perception skills. When the auditory system is compromised, two types of hearing disorders may occur: hearing loss and/or auditory processing disorder.^2^,^3^

The maturation process of the central auditory system structures starts as the child is exposed to sounds, mainly those of a linguistic nature, and ends at about 12 years of age, when auditory skills are fully developed.^3^

The hearing skills involved in central auditory processing and related to language development are described as follows: selective attention, sound detection, auditory discrimination, location, recognition, comprehension, memory, auditory closure, and auditory figure and ground.^4^

Auditory processing may become dysfunctional as a result of deprivation of acoustic experiences during the early stages of development; such deprivation may occur consequently to a number of factors, such as: sensorineural hearing loss, neuroplastic disorders, acoustic neuroma and others; conductive hearing loss; neurologic disorders arising from encephalopathies or secondary to infections such as meningitis and encephalitis.^5^,^6^

Other factors that may hamper auditory processing are: poor social and environmental conditions, medical/emotional problems, and drug abuse.^6^

Involved individuals present problems in oral communication, reading, and writing skills, cognitive functions such as attention and memory, and display learning disabilities, immature behaviors, and tendency to isolate themselves, thus hampering socialization.

Auditory processing disorders may be connected to etiologic agents that may cause injury to the central nervous system, such as illicit drugs.

More specifically, drugs change one's perceptions, mood, and sensations, thus inducing - even if temporarily - feelings of pleasure, euphoria, and relief from fear, pain, frustration, anxiety and others, as a result of their impact upon the central nervous system.^7^

Illicit drugs act mainly on the brain. They produce psychical alterations of variable quality and intensity depending on the type and amount of drug taken by the user. Other factors to take into account are the user's own specific individual characteristics, expectations over the drug taken, and the circumstances in which drugs are taken. It is important to note that illicit drugs also act upon other organs such as the heart, bowels, and blood vessels; their use, however, is more specifically connected to the effect they produce upon the user's central nervous system.^8^

Drugs can be categorized as licit and illicit. In Brazil, drugs deemed illicit are the ones whose trade and consumption are forbidden by Law, such as marijuana, crack cocaine, cocaine, heroin etc. Licit drugs, by their turn, can be freely marketed and consumed. A few of them are: nicotine, alcohol, and psychiatric medication.^9^

In this study, the concept of illicit drugs was applied to describe psychoactive substances taken through whatever means possible - orally, inhalation, intravenously etc - with the purpose of altering the function of user's central nervous system.

These alterations change the user's sense of perception and state of consciousness as such substances may stimulate, depress, or disturb the central nervous system.^9^

Some of the best known and more commonly used psychoactive substances are cocaine, marijuana, crack cocaine, lysergic acid diethylamide (LSD), and heroin.

Cocaine is an alkaloid extracted from coke leaves and other similar plants from the Amazon and Andean regions. The effects of cocaine are not consistent, and depend on the amount taken, bioavailability, and effect duration, which by its turn varies according to administration mode and user-specific traits.^10^ Cocaine is easily absorbed by mucosal tissue, and the mere contact with the powder produces a topical sensation of cool and anesthesia. Nasal aspiration provokes minor nose bleedings and mucosal irritation; prolonged repeated vasoconstriction leads to tissue necrosis and injuries such as atrophic rhinitis and even perforated septum with deep epistaxis.^11-I^ The drug acts on the central nervous system, more significantly on the cerebral cortex, stimulating and originating motor phenomena that may evolve to epilepsy-like seizures; it also changes one's psyche, producing subjective phenomena and effects such as excitement, euphoria, loquaciousness, sardonic laughter, and false sensations; these effects are however transient, as soon after depression, fatigue, numbness, and sleepiness set in.^12-I^

Marijuana is taken in the form of a cigarette made from the leaves, stem, fruit, and seeds of a plant called cannabis sativa, whose main active principle is tetrahydrocannabinol (THC). Users may experience hypersensitivity to sensorial stimulation and altered temporal perception. Marijuana's physiological effects are quite characteristic, such as conjunctival vessel dilation that leaves users with red eyes. Other possible effects are muscle weakness, fine hand tremor, and balance/gait alterations. Immunology studies have shown that marijuana dials down the body's natural defenses, changes chromosomes with genetic damage, alters hormone regulation, may produce temporary sterility and impotence, and harms the central nervous system.^11-II^ Intoxication by cannabis may alter cerebral function and introduce memory disorders, altered thinking and feelings of awkwardness, depersonalization, and hallucinations resulting from the direct impact the drug has on the central nervous system.^12_II^

Crack cocaine is not a new drug, but a new way of taking cocaine. It is obtained as the final product of cocaine is mixed with sodium bicarbonate and water, to be then heated and smoked from a pipe. It contains 75% pure cocaine; the narcotic effect of smoking crack cocaine is as fast as injected cocaine; it is a highly addictive and lethal drug.^13^

It is usually the first illicit drug users take as they start a history of addiction.^14^

LSD - lysergic acid diethylamide is considered to be the most potent drug. It is a psychomimetic or psycho-epileptic drug, as it may create a state in which there is a split in personality, simulating the symptoms observed in schyzophrenia.^15^

Heroin is obtained from morphine (the principal component in opium and one of the most powerful analgesic drugs); it is a white bitter powder. Narcotic effects are: numbness and dizziness, combined with feelings of lightheadedness and euphoria; users may also experience nausea and vomiting, but these symptoms disappear shortly. When dependence sets in, users need to inject heroin every four to six hours to avoid the inconveniences of abstinence, namely cramps, anxiety, generalized pain, lethargy, apathy, and fear. Excessive doses of heroin may put users in a coma. Heroin has been thought to produce alterations at a molecular level, as relapses are frequent and the desire to go back to the drug is quite strong and persistent.^15^

The impact of illicit drugs upon the central nervous system is devastating, as the CNS is an information processing entity. Impulses coming from all sensorial pathways arrive at the cerebral cortex for processing and interpretation.^16^

The cerebral cortex is divided into four regions or lobes, each playing a different role. The left frontal lobe is related to speech and writing; the left temporal and occipital lobes are connected to perception and comprehension of spoken and written language. The primary auditory cortex produces the ability to discriminate sound frequencies and intensities; it has a temporal pattern and is involved in the localization of sound sources, speech perception, and in the development of auditory processing skills and phonologic consciousness skills.^17^

Different sets of problems and different locations result in different types of auditory processing disorders.^18^

Studies describing neurophysiologic disorders in illicit drug users list the following as the most frequently observed infirmities: attention deficit, memory deficit, and impaired speech. These disorders are similar to the ones found in patients with CNS prefrontal and temporal disorders.^19^, ^20^, ^21^

Individual factors connected to larger contextual matters such as low income level, having drug users in the family and/or friend circles are among the most frequently mentioned reasons that led individuals to try drugs.^22^

Hearing comprises a complex neural network connected to the central auditory pathways; hearing skills require integral pathways to allow proper spoken stimuli processing.^23^

Hence comes the question as to by how much the use of illicit drugs could hamper central auditory processing. This paper aims to assess the selective attention hearing skills of subjects with a history of illicit drug use and check whether the time for which these individuals used drugs affects the degree of observed disorder.

## MATERIALS AND METHOD

This is a descriptive, observational, cross-sectional, statistical, comparative, retrospective study focused on diagnostic procedures. This study was approved by the Research Ethics Committee at our University under permit 001-AF/05. All subjects that took part in this study signed a Free Informed Consent Term.

Nineteen males with history of illicit drug use were enrolled in the study. All individuals were either admitted in a specialized institution for rehabilitation or were former drug users working for or providing support at this institution located in the countryside of São Paulo State, Brazil.

Only male subjects were selected as the elected institution did not offer accommodation for female individuals. Enrollment criteria: individuals free of peripheral hearing loss and able to consciously manifest their desire to participate in the study.

Materials: protocols adopted to capture subject interview and test findings. Equipment: HEINE mini 2000 otoscope; two-channel audiometer (AC-33 - earphone: TDH-39); Interacoustic AZ 7 electroacoustic impedance measurement device; SIEMENS AZP 7, 8, 9, 10, 11 probe; Madsen ZODIAC electroacoustic impedance measurement device; Aiwa CSD-A170 CD player; Compact disc (volume 2) to test auditory processing skills from the manual featured in Processamento Auditivo Central.^24^ All test devices were properly calibrated as described in standards ANSI S3.6- 1996/ISO-389-1991/ ISSO-8798/ANSI S3.43- 1992.

Data collection procedures were implemented in the following order:

1°) Analysis and authorization by the University Research Ethics Committee.
2°) Contact rehabilitation center.
3°) Invite subjects that meet enrollment criteria admitted at the institution and who could be transported to the facility where the tests would be conducted - Clinical Audiology Laboratory of a university located in the countryside of São Paulo State.
4°) Obtain participant signatures on Free Informed Consent Term.
5°) Carry out initial interview to gather information and describe the subject population based on the following parameters: age, time for which subjects have used drugs, drugs of choice, reason why subjects started doing drugs, and how long subjects have been drug-free (Chart 1, Chart 2, Chart 3, and Chart 4).

**Chart 1:** Participating subjects: age, educational level, age at which started taking drugs, time for which has used drugs, time for which has been drug-free, reasons why started taking drugs.

Subject	Age	Education	Started using	Time using drugs	Time free of drugs	Reasons
1	21y11m	5th grade	13 y/o	8 y 4 m	7 months	Drug trafficking
2	20y11m	9th grade	11 y/o	9 y 3 m	7 months	Inferiority complex
3	25y 9m	7th grade	19 y/o	5 y 9 m	12 months	Lack of God, character flaw
4	18y10m	5th grade	13 y/o	4 y 4 m	18 months	Curiosity, family
5	19y 4m	6th grade	13 y/o	5 y 5 m	9 months	Influenced by friends
6	47y 3m	5th grade	16 y/o	30y 6m	9 months	Curiosity
7	33y 3m	Incomplete secondary education	12 y/o	15y 3m	72 months	Influenced by friends
8	21y 9m	Incomplete secondary education	18 y/o	3y 6m	3 months	Influenced by friends, lack of God
9	26y 8m	Incomplete secondary education	14 y/o	12y 2m	6 months	Influenced by people, Curiosity
10	21 y	5th grade	16 y/o	4y 6m	6 months	Influenced by a girl
11	35y11m	Incomplete secondary education	15 y/o	20y 9m	2 months	Curiosity
12	15y11m	9th grade	10 y/o	5y 6m	5 months	Curiosity
13	16y11m	5th grade	9 y/o	7 y 7m	4 months	Influenced by friends
14	19y 8m	8th grade	13 y/o	6 y 6 m	2 months	Curiosity
15	26y 4m	Incomplete college	17 y/o	9 y	4 months	Curiosity
16	30y11m	9th grade	18 y/o	12y7m	4 months	Influenced by friends
17	28 y5m	5th grade	12 y/o	16y4m	1 month	Curiosity
18	25 y9m	5th grade	14 y/o	11y 6m	3 months	Curiosity
19	18 y	6th grade	12 y/o	5 y 9m	3 months	Curiosity

**Chart 2:** Drugs of choice.

Drug type and time for which used substance					
Subjects	Marijuana	Cocaine	Crack cocaine	LSD	Heroin
1	7 years	-------	2 y 6 m	-------	-------------
2	8 years	4 years	4 years	----------	4 years
3	2 years	1 a e 6m	1 y 6 m	---------	-----------
4	3 years	3 years	3 years	----------	------------
5	3 years	3 years	5 years	---------	----------
6	30 years	Did not know	4 years	--------	-----------
7	21 years	6 years	3 years	-----------	------------
8	2 years	1 year	2 years	-----------	----------
9	2 years	10 years	----------	----------	-------------
10	4 years	1 year	3 years	-----------	------------
11	5 years	2 years	1 year	----------	------------
12	4 years	3 years	2 years	2 years	-----------
13	4 years	-------	4 y 6 m	-----------	---------
14	1 a e 6 m	---------	5 y 6 m	----------	---------
15	8 years	6 years	6 years	6 years	----------
16	12 years	2 years	2 years	----------	---------
17	16 years	8 years	8 years	----------	----------
18	11 years	2 years	2 years	----------	------
19	5 years	5 years	1 year	-----------	-----------
Mean	8 y 1 m	4 y 1 m	3 y 3 m	4 years	4 years

**Chart 3:** Drug combinations used by subjects in the sample.

Drug combinations	Marijuana Crack	Marijuana Crack Cocaine	Marijuana Crack Cocaine LSD	Marijuana Crack Cocaine Heroin
Number and percent of subjects	03 – 16%	13 – 68.5%	02 – 10.5%	01 – 05 %

**Chart 4:** Subject mean age, mean age when started taking drugs, mean time of substance abuse, and mean time of abstinence.

Mean age		Mean time	
Subject	When started taking drugs	Substance abuse	Abstinence
25 y 04 m	13 y 04 m	10 y 07 m	14 months

Legend: y equals; years m equals; months
6°) Perform ENT examination to rule out peripheral auditory system disorders that could possibly prevent the assessment of hearing test results.
7°) Conduct audiological examination (tone threshold audiometry, logoaudiometry, impedance test) to find subject auditory thresholds; assess the degrees of speech reception and recognition; look into middle ear status. Normal audiological test results are required for subjects to undergo auditory processing assessment.
8°) Auditory processing assessment through SSW (Staggered Spondaic Word) test standardized for Portuguese.^25^

All nineteen subjects selected in our sample had normal audiological test results. Mean group age was 20 years and four months. Sixteen individuals started taking illicit drugs when they were under 15 years of age. Marijuana was the first drug tried my most subjects; it was later used in combination with other drugs, as shown in Chart 3. As also seen in Chart 3, 80% of the subjects took three or more illicit drugs concurrently.

The number of errors verified in the four listening conditions presented during the test (right non-competitive; right competitive; left competitive; left non-competitive) were compared in the analysis of the SSW test results, as proposed in the literature.^27^ This approach was adopted due to the difficulty in standardizing a control group made up by truly non-drug users based only on interview findings and no pharmacologic tests.

The data set used as comparison parameter comprised the performance of individuals with normal hearing in the Portuguese SSW test in terms of the number of errors made in each condition. The author assessed one-hundred audiologically normal individuals with ages ranging between 18 and 39 years with at least complete primary education. Fifty individuals were females and fifty were males.^26^

The results on the total number of errors observed in each listening condition were compared against the results obtained by the first nineteen male subjects with normal hearing.^26^ These test results were selected based on similarities between the subjects in this sample and the individuals selected in the study (age range, gender, and level of education).

The results for the nineteen subjects included in the group used as a comparison parameter^26^ made up the control group (CG), while the results for the subjects included in the study sample made up the research group (RG).

The total number of errors between both samples was analyzed based on the summation of the total number of errors observed in all four listening conditions (right non-competitive; right competitive; left competitive; left non-competitive). Then, the results obtained from this analysis were compared for both control and research groups and submitted to statistical analysis.

Comparison between groups aimed at verifying whether there was any statistically significant difference on the number of errors made by the drug user group in comparison to the group of subjects with normal hearing as described in the literature.^26^

SSW test results were not characterized as normal or altered, as such type of analysis would not contemplate the possibly deleterious effect drugs could have on the central nervous system and listening skills.

The case group was subdivided into two subgroups to check for possible connections between the time for which subjects had been using drugs and their performance on the SSW test. Research subgroup I contained individuals who had used drugs for 4 to 8 years, while research subgroup II featured subjects who had used drugs for 8 years and a month to 30 years and six months. Analysis done on subgroups I and II verified whether time for which the subjects had done drugs was a relevant variable in relation to the number of errors observed in each listening condition.

The Mann-Whitney test was performed as it is suitable to compare two sets of numeric data from independent samples and no assumptions were intended around the distribution of the data.

## RESULTS

As results are presented, the group referred to as ‘control’ depicts data extracted from the literature26, while the error ratings observed for the subjects targeted by this study are under the title ‘research group.’

Chart 5 presents the analysis of the results of the SSW test for both groups (control and research) and each of the group members.

**Chart 5:** Number of errors made by control group (normal hearing) subjects and research group individuals in non-competitive and competitive listening conditions.

Control Group					Research Group				
Subjects	RNC	RC	LC	LNC		RNC	RC	LC	LNC
S1	0	0	0	0	S1	5	8	9	3
S2	0	1	1	0	S2	2	8	2	1
S3	0	1	0	0	S3	3	6	5	4
S4	0	2	1	0	S4	5	5	11	2
S5	0	0	0	0	S5	1	0	2	0
S6	1	1	1	1	S6	1	2	2	0
S7	0	0	0	0	S7	0	4	1	0
S8	0	0	1	0	S8	3	5	1	1
S9	0	1	0	0	S9	0	14	12	1
S10	0	0	0	0	S10	2	10	7	1
S11	0	1	2	1	S11	3	1	3	1
S12	0	1	1	0	S12	2	1	0	1
S13	0	2	1	0	S13	1	0	2	0
S14	0	0	0	0	S14	1	8	2	0
S15	0	0	0	0	S15	1	2	0	0
S16	0	0	1	0	S16	1	3	0	0
S17	0	0	1	0	S17	1	4	3	0
S18	0	0	1	0	S18	4	8	4	3
S19	1	0	0	0	S19	1	1	2	1

Research and control group results were compared in terms of the summation of the total number of errors and mean percent errors for each group in all four listening conditions. Chart 6 shows this comparison.

**Chart 6:** Comparison between total number of errors and percent mean values for control and research groups in non-competitive (RNC and LNC) and competitive (RC and LC) conditions.

Total of errors					Percent mean value of total errors			
Subjects	RNC	RC	LC	LNC	RNC	RC	LC	LNC
Control Group	2	10	11	2	0.10	0.52	0.57	0.10
Research Group	37	90	68	19	1.94	4.73	3.57	1

Subjects in the research group were subdivided into two groups to find whether the time for which individuals had been using drugs had any impact on the total number of errors observed. Research subgroup I contained individuals who had used drugs for 4 to 8 years, while research subgroup II featured subjects who had used drugs for 8 years and a month to 30 years and six months. Chart 7 shows the comparison results.

**Chart 7:** Comparison between total number of errors and percent mean values for research subgroup I (used drugs for less time) and research subgroup II (used drugs for longer).

Total of errors					Mean error value			
Subjects	RNC	RC	LC	LNC	RNC	RC	LC	LNC
Research SG I – 4y-8y	19	36	32	10	2.11	4	3.55	1.11
Research SG II-8y/30y6m	18	52	36	9	1.8	5.2	3.6	0.9

Comparative analysis of mean number of errors made by research group individuals is shown in Table 1, Table 2. Table 1 shows an extremely significant statistical difference (p< 0.001) when research and control groups are compared in ‘right non-competitive’ and ‘right competitive’ conditions. In ‘left competitive’ and left non-competitive' conditions the statistical difference was quite significant (p< 0.01).

**Table 1:** Statistical analysis for research and control groups

		Research group				Control group		
	RNC	RC	LC	LNC	RNC	RC	LC	LNC
Mean	1.947	4.737	3.579	1.000	0.1053	0.5263	0.5789	0.1053
Standard deviation	1.508	3.827	3.626	1.202	0.3153	0.6967	0.6070	0.3153
Minimum confidence interval	1.220	2.892	1.831	0.4207	0.04671	0.1905	0.2864	0.04671
Maximum confidence interval	2.674	6.582	5.327	1.579	0.2572	0.8621	0.8715	0.2572

**Table 2:** Statistical analysis for research subgroups I and II

		Research subgroup I				Research subgroup II		
	RNC	RC	LC	LNC	RNC	RC	LC	LNC
Mean	2.111	4.000	3.556	1.111	1.800	5.400	3.600	0.9000
Standard deviation	1.364	3.674	3.504	1.269	1.687	4.033	3.921	1.197
Minimum confidence interval	1.062	1.176	0.8622	0.1354	0.5935	2.515	0.7947	0.04355
Maximum confidence interval	3.160	6.824	6.249	2.087	3.006	8.285	6.405	1.756

The data shown on Table 2 did not present statistically significant differences (p< 0.05) in the ‘right non-competitive’, ‘right competitive’, and left non-competitive' listening conditions when subjects on subgroups I and II were compared (both from the research group). However for left competitive' listening, statistically significant differences were found although numerically the difference is small; this can be explained by the variability between groups.

## DISCUSSION

The central auditory system is fully developed in human beings around the age of 12, when listening skills are completely evolved.^3^ Considering the interviews more carefully, we saw that eleven individuals started taking illicit drugs when they were under 15 years of age, a period in which the central auditory system, the brain hemispheres and the corpus callosum are still undergoing anatomic and physiological development. Any harm perpetrated against these structures and functions may impair the neural development process.

The interviews have also shown that curiosity and influence from friends were the main factors behind the subjects choosing to take drugs for the first time. This finding is widely reported in the literature and described alongside other motivational aspects such as individual traits, emotional/affective problems, users in the family, lower income level, combined with 'deviant' friends.^9^,^22^

When comparing the SSW test results, we verified that the number of errors in the four listening conditions for research and control groups had extreme statistically significant differences in 'right non-competitive' and 'right competitive' conditions. In 'left competitive' and 'left non-competitive' statistical differences were quite significant. These differences confirm that the negative impact drugs have on the central nervous system also adversely affects the auditory portion of the system. Once there is no way to prove the existence of auditory processing disorders before subjects started taking drugs, we may state that the negative effects drugs had on the central auditory system triggered or worsened already existing disorders.

Central auditory processing skills, cognitive skills, problem resolution skills, attention, and verbal fluency rely on the integrity of the central nervous system; such integrity can be adversely impacted by a number of factors, among them licit and illicit substances, as observed in this study and in other reports in the literature.^19^,^21^

Drugs act intensely and hazardously upon the central nervous system, and quite significantly upon the cerebral cortex, thus supporting the possibility that drugs can harm the central auditory system.^6^,^9^,^12^

No significant increase in the number of errors in the SSW test was observed as a function of longer periods of substance abuse. This finding is explained by the fact that dependence is related to risk factors connected to bioavailability, social context, and family environment, all of which highly variable from person to person as described in the literature.^9^,^10^,^22^

## CONCLUSION

The results reported in this study show that the deleterious impact drugs have on the central nervous system significantly affects the auditory system, triggering central auditory processing disorders or worsening pre-existing disorders. No worsening in listening skills was however seen as a function of longer periods of substance abuse.

The Portuguese version of the Staggered Spondaic Word test is a valid listening skill assessment procedure in the investigation of the effects substance abuse may have on the central nervous system.

The findings reported in this study make evident the need for further clarification and characterization of the relationship between substance abuse and central auditory system disorders, looking at improving the development of procedures to enhance the rehabilitation offered to former drug users.
